# Unveiling astrocytic control of cerebral blood flow with optogenetics

**DOI:** 10.1038/srep11455

**Published:** 2015-06-16

**Authors:** Kazuto Masamoto, Miyuki Unekawa, Tatsushi Watanabe, Haruki Toriumi, Hiroyuki Takuwa, Hiroshi Kawaguchi, Iwao Kanno, Ko Matsui, Kenji F. Tanaka, Yutaka Tomita, Norihiro Suzuki

**Affiliations:** 1 Faculty of Informatics and Engineering, University of Electro-Communications, 1-5-1 Chofugaoka, Chofu, Tokyo 182-8585, Japan; 2 Brain Science Inspired Life Support Research Centre, University of Electro-Communications, 1-5-1 Chofugaoka, Chofu, Tokyo 182-8585, Japan; 3 Molecular Imaging Centre, National Institute of Radiological Sciences, 4-9-1 Anagawa, Inage, Chiba 263-8555, Japan; 4 Department of Neurology, Keio University School of Medicine, Shinanomachi, Shinjuku, Tokyo 160-8582, Japan; 5 Department of Neuropsychiatry, Keio University School of Medicine, 35 Shinanomachi, Shinjuku, Tokyo 160-8582, Japan; 6 Division of Interdisciplinary Medical Science, Tohoku University Graduate School of Medicine, 2-1 Seiryomachi, Aoba, Sendai, Miyagi 980-8587, Japan; 7 Tomita Hospital, 32 Minaminakamachi, Motojuku-cho, Okazaki, Aichi 444-3505, Japan

## Abstract

Cortical neural activities lead to changes in the cerebral blood flow (CBF), which involves astrocytic control of cerebrovascular tone. However, the manner in which astrocytic activity specifically leads to vasodilation or vasoconstriction is difficult to determine. Here, cortical astrocytes genetically expressing a light-sensitive cation channel, channelrhodopsin-2 (ChR2), were transcranially activated with a blue laser while the spatiotemporal changes in CBF were noninvasively monitored with laser speckle flowgraphy in the anesthetised mouse cortex. A brief photostimulation induced a fast transient increase in CBF. The average response onset time was 0.7 ± 0.7 sec at the activation foci, and this CBF increase spread widely from the irradiation spot with an apparent propagation speed of 0.8–1.1 mm/sec. The broad increase in the CBF could be due to a propagation of diffusible vasoactive signals derived from the stimulated astrocytes. Pharmacological manipulation showed that topical administration of a K^+^ channel inhibitor (BaCl_2_; 0.1–0.5 mM) significantly reduced the photostimulation-induced CBF responses, which indicates that the ChR2-evoked astrocytic activity involves K^+^ signalling to the vascular smooth muscle cells. These findings demonstrate a unique model for exploring the role of the astrocytes in gliovascular coupling using non-invasive, time-controlled, cell-type specific perturbations.

## Introduction

Astrocytes are the brain cells that form an interface between the brain vasculature and central nervous system. In response to neural requirements, astrocytes control the vascular tone, the mechanisms of which have been extensively studied in both brain slices *in vitro*^1^,^2^ and animal brains *in vivo*^3^. Because dysfunction of the astrocytic control of the cerebrovasculature is involved in the pathogenesis of several neurodegenerative disorders^4^, the underlying mechanisms for controlling the cerebrovascular dynamics have received a great deal of attention in relation to preventing or ameliorating neurovascular dysfunctions.

The major pathway for regulating cerebrovasculature is currently thought to involve the release of glutamate through locally evoked neurons, leading to either vasodilation or vasoconstriction depending on astrocyte-mediated mechanisms^5^,^6^. Metabotropic glutamate receptors (mGluR5 and 1) in astrocytes are believed to initiate the intracellular Ca^2+^ elevation that leads to the release of vasoactive molecules at the endfeet near the vascular smooth muscle cells^1^,^2^. Large-conductance Ca^2+^-sensitive K^+^ (BK) channels expressed in astrocytes have also been shown to play a key role in determining the vascular tone^7^,^8^. Early studies assumed that the intracellular Ca^2+^ elevation in the astrocytes is a prerequisite for altering the vascular tone^9^,^10^,^11^. However, whether the astrocytic Ca^2+^ elevation in response to neural activity exclusively triggers rapid vasodilation is highly debated^12^,^13^,^14^,^15^,^16^,^17^,^18^,^19^. Selective antagonists of mGluR5 had no detectable effects on the hemodynamic response to sensory stimulation^13^. The developmental changes in the mGluR expression profile were also noticed in the mouse cortical astrocytes^20^. Furthermore, several studies demonstrated that a genetic deletion of the cytosolic Ca^2+^ increase in the astrocytes has no effect on the neurovascular coupling^15^,^16^,^17^,^18^. One recent study revealed that Ca^2+^ transient in the astrocytic process, but not soma, triggers a rapid hemodynamic response in the nearby vasculatures^19^. However, the source of the Ca^2+^ transient remains undetermined.

To study the astrocytic role in isolation, specific manipulation of astrocytic activity is required. Caged Ca^2+^ can be loaded into astrocytes, and astrocytes can be stimulated by the uncaging of these compounds. However, astrocyte specific loading is often not warranted. Specific injection of current into astrocytes via patch electrodes has been conducted^5^; however, given the low impedance of the cells, somatic depolarisation will likely not propagate to the fine processes where the cells make contact with the vessels. In both cases, invasive methods are required to stimulate astrocytes, which are known to rapidly change their properties in response to invasive stimuli^21^; thus, the function of these cells in their natural state is difficult to discern.

In the present study, we utilised a recently developed mouse with genetically targeted expression of a light-gated cation channel, channelrhodopsin-2 (ChR2)^22^, in astrocytes^23^. Optogenetics is an ideal tool for accomplishing non-invasive, time-controlled, cell-type specific perturbation of astrocytic activity in the *in vivo* brain (for reviews^24^,^25^). With this animal model, previous studies demonstrated that astrocytic activity is specifically enhanced by photostimulation of the ChR2-expressing astrocytes^23^, leading to synaptic plasticity and perturbation of cerebellum-modulated motor behaviour^26^. Photostimulation of the ChR2-expressing astrocytes causes a transient depolarisation of the membrane potential, and this effect is reversible and reproducible^23^,^26^.

**Figure 1:**
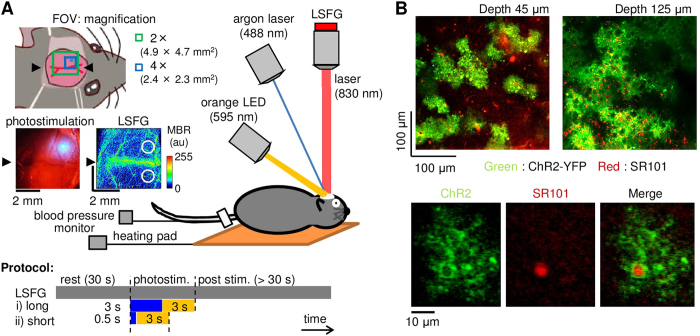
**A**) Experimental setup (original drawing). To open ChR2 channels specifically expressed in the astrocytes, an argon laser was induced through an electromagnetic shutter, while spatiotemporal CBF were non-invasively monitored with laser speckle flowgraphy (LSFG), which consisted of an excitation infrared laser and a detection camera (CCD) attached to a microscope. To close the channels, an orange LED was also irradiated following the cessation of the blue laser irradiation. The field of view (FOV) for the LSFG was either 4.9 mm × 4.7 mm or 2.4 mm × 2.3 mm with an objective lens of 2 × (green square) or 4 × (blue square), respectively. A representative spatial arrangement of the irradiated laser spot (0.5 mm in diameter) and a baseline image of the LSFG are shown in bright field and mean blur rate (MBR) images, respectively. A colour bar represents the 8-bit signal level of the MBR. Two circles (1 mm in diameter) in the LSFG image represent the locations of the regions of interest used for calculation of the photostimulation-induced changes in CBF in the ipsilateral and contralateral cortices. Two types of photostimulation were tested: i) long blue laser irradiation (3 sec) followed by a 3-sec orange LED irradiation and ii) short blue laser irradiation (0.5 sec) followed by 3-sec orange LED irradiation. **B**) Representative images of ChR2-expressing astrocytes (green) co-labelled with sulforhodamine 101 (SR101; red) captured at depths of 45 μm (top left) and 125 μm (top right) from the cortical surface of the extracted brain with two-photon microscopy. The enlarged view (bottom) represents co-localisation (right) of the ChR2-expressing fine processes (left) with the SR101-positive astrocytic soma (middle).

Here, we tested whether optogenetic activation of ChR2-expressing astrocytes could influence local cerebral blood flow (CBF). Because the dynamic properties of cerebrovasculature are sensitive to mechanical insults, such as that generated by an invasive surgical procedure used to directly measure vessel diameters, we performed a non-invasive imaging of spatiotemporal CBF as an indirect assessment of the vascular dynamics using laser speckle flowgraphy (LSFG) through the intact skull (Fig. 1A). The ChR2 expression in the cortical astrocytes were ensured by labelling them with sulforhodamine 101 (SR101) after the experiments (Fig. 1B). Because photostimulation of the ChR2-expressing astrocytes may lead to the release of glutamate and potassium ions^26^, pharmacological tests were further conducted to determine the signalling involved in the photostimulation-induced changes in CBF. The glutamate released from the activated ChR2-astrocytes could stimulate the neighbouring neurons and/or astrocytes, leading to vasodilation via activation of cyclooxygenase (COX)-derived prostaglandin pathways^27^,^28^. The involvement of this pathway was tested with topical application of tetrodotoxin (TTX) or a non-selective COX inhibitor, sodium indomethacin, applied to the stimulated cortex^3^,^29^. An increase in the extracellular concentration of K^+^ may induce a rapid vasodilation via an activation of K^+^ channels in the vascular smooth muscle cells^30^,^31^. To inhibit this K^+^ signalling, we topically applied BaCl_2_, an inward rectifier K^+^ (K_ir_) channel blocker, to the stimulated cortex.

## Results

### Photostimulation of ChR2-expressing astrocytes induced a rapid increase in the CBF

**Figure 2:**
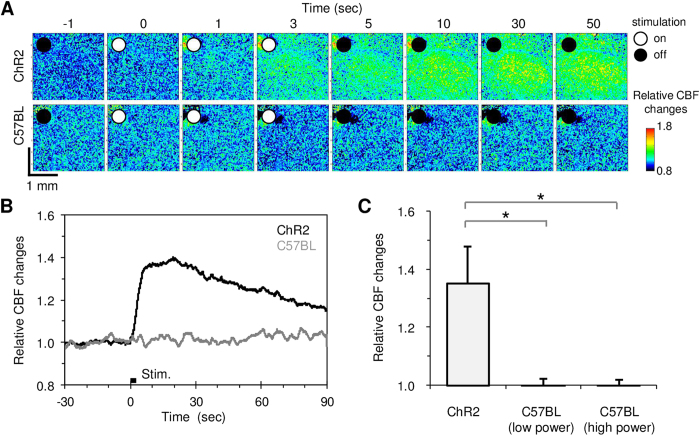
Photostimulation induced CBF changes. **A**) Baseline and activation-induced CBF images captured with LSFG. A robust increase in CBF was evoked only in the ChR2 mouse (top) and not in the C57BL/6 mouse (bottom). White and black circles in the image indicate the presence or absence of a 3-sec blue laser irradiation, respectively. The signal intensity was normalised to the baseline level of the mean blur rate (MBR). **B**) Average time course of the photostimulation-induced CBF changes in the ChR2 (N = 7) and C57BL/6 mice (N = 4). **C**) Comparison of the photostimulation-induced magnitude of the CBF changes. The photostimulation evoked an increase in CBF in the stimulated cortex of ChR2 mice but not in C57BL/6 mice, even at a higher power of the blue laser. A significant increase in CBF (*P < 0.05) was detected in the ChR2 mice compared to the C57BL/6 mice.

We observed that a brief photostimulation with blue laser irradiation (0.1–0.3 mW) of ChR2-expressing astrocytes induced a rapid increase in the CBF (Fig. 2A–B). The increased CBF gradually returned to its baseline level, and this transient response to photostimulation was reproducible (Supplementary Figure S1). The average response magnitude of the CBF increase evoked at the activation foci (1 mm in diameter) was 35 ± 13% relative to the pre-stimulus baseline in the ChR2 mice (N = 7). We confirmed that neither low (0.1–0.3 mW) nor high (2.8–3.3 mW) power irradiation of the blue laser affected the CBF in normal C57BL/6J mice (N = 4; Fig. 2C). The results indicate that laser irradiation alone does not evoke changes in CBF in our experimental conditions. In addition, no measurable changes in the animal physiology (systemic blood pressure and heart rate) upon photostimulation were observed.

### Wide spread increase of photostimulation-induced CBF

**Figure 3:**
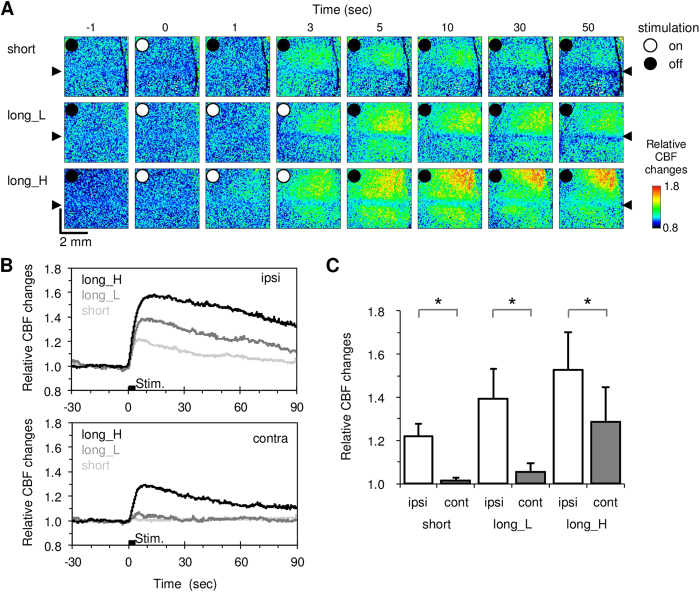
Comparison of the photostimulation-induced CBF changes in the stimulated ipsilateral and unstimulated contralateral cortices in ChR2 mice (N = 10). **A**) Spatiotemporal dynamics of the CBF responses to short irradiation (top), long blue laser irradiation with low power (long_L, middle), and long blue laser irradiation with high power (long_H, bottom). Immediately after irradiation with the blue laser at time 0 (white circle), the CBF increased in the stimulated cortex. Weak stimulation evoked localised increases in the CBF, while greater stimulation provoked robust and prolonged increases in the CBF. **B**) Average time courses of the CBF responses for ipsilateral ROIs (top) and contralateral ROIs (bottom). **C**) Average response magnitude. A significant increase in the CBF was detected in the ipsilateral cortex compared to the contralateral cortex (*P < 0.05).

We next compared the CBF responses in the laser-irradiated ipsilateral and unirradiated contralateral cortex in the same ChR2 mouse (N = 10). The average response magnitude evoked with a 0.5-sec short blue laser irradiation was 22 ± 6% relative to the baseline in the ipsilateral cortex, but it was negligible in the contralateral cortex (Fig. 3A,B). A longer blue laser irradiation (3.0 sec) evoked greater responses in both the ipsilateral and contralateral cortices (Fig. 3A,B). For all photostimulation levels tested (0.5-sec low power, 3-sec low and high power blue laser irradiation), statistically significant differences in the CBF response magnitude were observed between the irradiated and unirradiated cortices (*P* = 2.8 × 10^−6^, 6.8 × 10^−6^ and 1.9 × 10^−4^, respectively, paired t-test; Fig. 3C).

**Figure 4:**
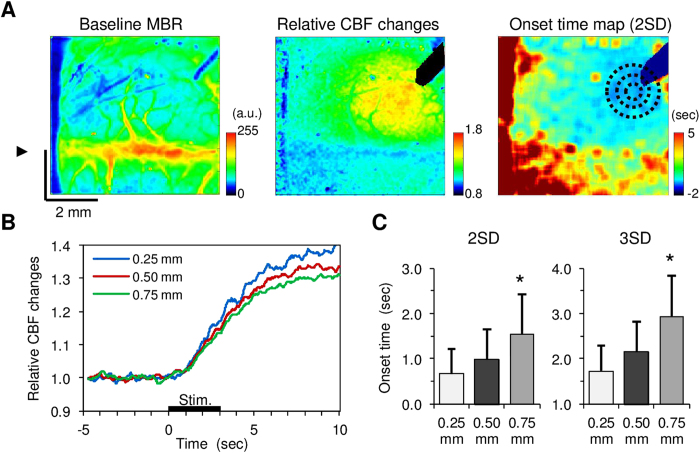
Spatiotemporal dynamics of CBF responses to transcranial photostimulation in ChR2 mice (N = 10). **A**) A representative image of the mean baseline MBR averaged for 10-sec pre-stimulus periods (*left*). The arrowhead indicates the location of the midline. Broad CBF changes were evoked with photostimulation (*centre*). A rapid increase in CBF was observed around the irradiation spot while the CBF onset spread from the activation foci (*right*). Different size ROIs (0.50 mm, 1.0 mm, and 1.5 mm in diameter; black dot circles) were placed in a concentric fashion at the activation centre. **B**) Average time course of the CBF responses measured at distances of 0.25 mm, 0.50 mm, and 0.75 mm from the central point of the activation. **C**) Mean onset time for CBF responses measured at distances of 0.25 mm, 0.50 mm, and 0.75 mm from the central point of the activation. Two different thresholds (2-fold and 3-fold standard deviations (SD) of the 10-sec baseline fluctuations) were applied to calculate the onset time of the CBF responses (left and right, respectively). A significant difference in the CBF response onset time compared to the activation foci was consistently found at a distance of 0.75 mm (*p < 0.05).

The average onset time of the CBF response at the ipsilateral activation foci for 3-sec blue laser irradiation was 0.7 ± 0.7 sec with a cut-off threshold of 2-folds standard deviation (SD) and 1.7 ± 0.9 sec for a cut-off threshold of 3-folds SD, which appeared to widely extend to the surrounding areas (Fig. 4A,B). A significant delay in the onset time was found at a distance of 0.75 mm (*p* < 0.05, Dunnett’s test) relative to the activation foci (Fig. 4C). Consequently, an apparent propagation speed measured from pixels 0.25-mm to 0.75-mm from the activation centre was 1.1 ± 0.7 mm/sec and 0.8 ± 0.5 mm/sec at a cut-off threshold of 2-fold and 3-fold SD, respectively. The mean activation area induced by all photostimulations tested was 3.9 ± 1.8 mm^2^, which was 20-fold larger than the irradiation spot of blue laser (0.2 mm^2^). These results indicate that widely spreading increase in the CBF could be due to a propagation of diffusible vasoactive signals derived from the stimulated astrocytes.

### Photostimulation-induced CBF changes involve activation of vascular K^+^ channels

**Figure 5:**
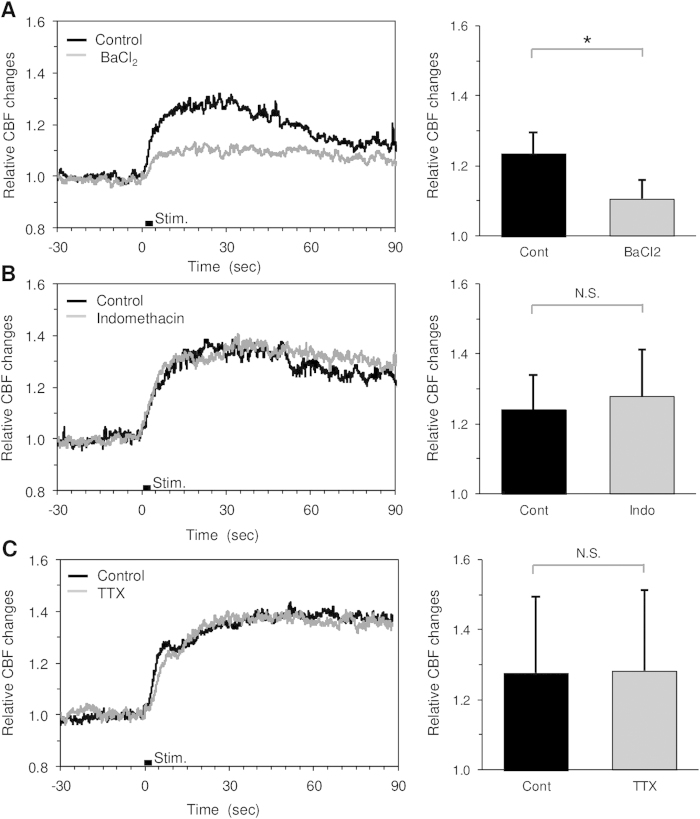
CBF responses to direct photostimulation measured before and after pharmacological treatment in ChR2 mice. **A**) Topical administration of BaCl_2_ (0.1–0.5 mM) significantly reduced the CBF responses (N = 6). **B**), **C**) Neither significant difference in the CBF response was detected for topical administration of indomethacin (Indo; 0.5 mM, N = 4) nor TTX (20–50 μM, N = 4).

Pharmacological manipulation showed that the CBF responses to photostimulation were significantly reduced by topical administration of BaCl_2_, but not indomethacin and TTX (Fig. 5). The magnitude of the CBF responses before and after administration of BaCl_2_ were 24% ± 6% and 11% ± 5%, respectively (*P* = 0.014, N = 6; Fig. 5A). In contrast, significant differences in the CBF response were neither detected for the treatment with indomethacin (24% ± 10% and 28% ± 14%, *P* = 0.29, N = 4; Fig. 5B) nor TTX (28% ± 22% and 28% ± 23%, *P* = 0.43, N = 4; Fig. 5C). We also observed that topical administration of BaCl_2_ on the exposed cortex had no detectable effects on the baseline neural activity and CBF measured with electroencephalography (EEG) and LSFG, respectively (Supplementary Figure S2, N = 4 ChR2 mice). These findings indicate that the photostimulation-induced CBF changes mainly involve direct K^+^ signalling to vascular cells.

## Discussion

The present study shows that photostimulation of the ChR2-expressing astrocytes evokes rapid and robust increases in CBF. In contrast to traditional tools that use invasive patch electrodes or caged compounds, the technique used here has great advantages in its methodology in which we specifically activate cortical astrocytes noninvasively. As this model allows manipulation of the local CBF by stimulating a specific brain site, it could be used as a unique tool to potentiate the CBF on demand. The CBF can be manipulated by different existing methods with pharmacological and/or respiratory perturbations; however, the optogenetic method has advantages because it does not affect the systemic circulation and global brain CBF, and thus effects originating from other body/brain regions would not complicate measurements. Combining the noninvasive manipulation and imaging of regional CBF therefore allows for region to region comparisons of the long-term neurovascular interactions in both healthy and neurovascular dysfunction models, such as evoked during aging, cerebral ischemia, and neurodegenerative diseases.

To noninvasively monitor the spatiotemporal CBF, we used LSFG which primarily reflect motion of red blood cells in the vessels^32^. Because LSFG is sensitive to any motions associated with induction of stimulation, other factors (such as swelling of the cells in the stimulated tissue) could also cause artificial signal changes. However, this possibility is unlikely because we observed that photostimulation to the astrocytes evoked vasodilation in the cortex (Supplementary Figure S3), indicating that the photostimulation of ChR2-expressing astrocytes causes vasodilation around the activated astrocytes and leads to rapid and broad increases in CBF. The tentative mechanisms involved in the vasodilation induced by the astrocytic activation with photostimulation are i) an increase in the K^+^ efflux through an open of ChR2 leading to a local change in the astrocytic membrane potential^26^ and/or ii) a change in the intracellular pH^33^.

We observed that a topical application of indomethacin (a non-selective COX inhibitor) had no measurable effects on the CBF response to photostimulation (Fig. 5B). Because a COX synthesis is involved in the astrocytic vasodilation via an intracellular Ca^2+^ elevation of the astrocytes^3^, our results indicate that the intracellular Ca^2+^ signalling may not play a major role in the photostimulation-induced CBF responses. A previous study with ChR2-expressing astrocytes also demonstrated that the astrocytic response to photostimulation in the cerebellum involves the release of glutamate, resulting in modulation of synaptic plasticity^26^. However, this observation doesn’t necessarily indicate that photostimulation to the cortical astrocyte also causes the release of glutamate in our experimental conditions. Glutamate is a potent vasodilator that act on the vascular cells via glutamate-sensitive cells but not a direct uptake by the vascular cells^34^,^35^, and the astrocytic release of glutamate could evoke neural responses. However, we observed no detectable effects of neuronal suppression with TTX on the CBF response to photostimulation (Fig. 5C). The findings therefore indicate that the photostimulation-induced CBF increase is directly driven by the astrocytic signalling to the vascular cells.

A variety of K^+^ channels are expressed in the vascular smooth muscle cells, and the activation of these channels results in a rapid dilation of the cerebral arteries^36^. We found that the average onset time of the CBF increase in response to astrocytic activation was 0.7 ± 0.7 sec after the onset of photostimulation (Fig. 4), which is within the range of previously reported values of the onset time examined for the CBF responses to physiological sensory stimulation^37^. Furthermore, topical application of BaCl_2_, a potent inhibitor of K_ir_ channels, significantly reduced the photostimulation-induced CBF responses (Fig. 5A). The findings indicate that astrocytic activity can lead to a rapid vasodilation driven by direct K^+^ release from the endfoot and/or other fine processes. Assuming that the diffusion coefficient of K^+^ in saline is 2 × 10^−9^ m^2^/sec^38^, it takes approximately 1 sec for K^+^ to reach a 60-μm distance in the brain tissue, which is far slower than the apparent propagation speed of the CBF response onset observed in the present study (0.8–1.1 mm/sec). The propagation speed of the astrocytic calcium wave induced by spreading depression was 35 μm/sec^39^, which is also slower than that observed in the present study. In contrast, a fast propagation of the vasodilation (2.4 mm/sec) along the pial artery was reported for sensory stimulation-induced vasodilation^40^. A rapid conduction of the vasomotion response (approximately 1 mm/sec) was also shown in the isolated cerebral arteries^41^. Considering these observations, we speculate that the rapid propagation of the CBF response to astrocytic activation observed in the present study involves i) astrocyte-to-astrocyte communications and/or ii) electrical conduction through the vascular cells.

The involvement of the K^+^ signalling to vascular cells evoked by astrocytic activation seems to be consistent with the earlier prediction proposed by Paulson and Newman^42^, which states that astrocytic release of K^+^ at the endfeet near the vasculature provokes a rapid vasodilation, rather than a diffusion of K^+^ into the extracellular space. In a later study, however, the hypothesis of K^+^ siphoning in neurovascular coupling was dismissed by Metea *et al.* (2007), who showed a negligible contribution of astrocytic depolarisation, via patch electrodes and elimination of astrocytic K_ir_ channels (knockout of K_ir_4.1 expressed in the astrocytes), to the nearby vascular tone^43^. Due to the low impedance of the cells, somatic depolarisation might not be sufficient to cause depolarisation at the foot process. Additionally, deletion of the K_ir_4.1 channels does not necessarily mean that K^+^ signalling to the vascular cells was exclusively deleted. Other K^+^ channels may contribute to the K^+^ signalling in the cells. An involvement of the voltage-dependent K^+^ (K_v_) channel and BK channel activations or the gap junction pathways would be interesting to be examined in relation to the astrocyte-to-astrocyte communication in the photostimulation-induced CBF response.

## Methods

### Animal preparations

Animal use and experimental protocols (No. 09058) were approved by the Animal Ethics Committee of Keio University Medical School, and all experimental procedures were in accordance with the university’s guidelines for the care and use of laboratory animals. A total of 21 male and female Mlc1-tTA::tetO-ChR2(C128S)-EYFP double transgenic mice (10–30 weeks, 32 ± 5 g) were used for the experiments. In these mice, C128S-modified ChR2 fused with EYFP was specifically expressed in astrocytes under the control of the Mlc1 promoter^23^. ChR2(C128S) is opened by blue light, and it requires yellow/orange light to close. As a control experiment, four normal male C57BL/6J mice (8 weeks, 21–25 g) were also subjected to repeated photostimulation to examine potential artefacts on the cortical microcirculation due to light irradiation. Under urethane anaesthesia (1.1 g/ kg, i.p.), the skull over the somatomotor cortex of both hemispheres was exposed. The body temperature was maintained at 36 °C with a heating pad (BWT-100, Bioresearch Centre Co. Ltd., Nagoya, Japan), and the systemic blood pressure and heart rate were monitored with a non-invasive blood pressure monitor (MK-2000ST, Muromachi Kikai Co. Ltd, Tokyo, Japan) at the tail, as needed.

### Photostimulation

For comparisons of the photostimulation-induced CBF changes between normal C57BL/6J mice and ChR2 mice, a 488-nm argon laser (CVI Melles Griot, Carlsbad, CA) supplied through an optical fibre and a beam collimator (FBC-203S, Neoark, Tokyo, Japan) was used to irradiate a spot at the centre of the parietal bone. The irradiation spot on the skull was 0.50 ± 0.02 mm in diameter (i.e., 0.2 mm^2^), which was captured with CCD camera (480 × 640 pixels with a pixel resolution of 35 μm/pixel). The irradiation image was converted to a binary image with applying a threshold at a half maximum of the pixel intensity in the image. The irradiation area was then approximated with a circle shape, and the diameter of the circle was measured. The mean irradiation power was measured with an optical power meter (8230 ADCMT, ADC, Tokyo, Japan). The duration of the laser irradiation was 0.5 sec (short) or 3 sec (long), which was regulated with an electromagnetic shutter (F77-7, Suruga Seiki, Shizuoka, Japan), and the intensity of the irradiation was adjusted using an ND filter placed in the optical path. Following cessation of the laser irradiation, a 595-nm LED light (0.1–0.3 mW, LEDP_HB01-A, Doric Lenses, Quebec, Canada) was used to irradiate the skull for 3 sec to close ChR2(C128S) channels (Fig. 1A). These sequential events were controlled with a pulse generator (Master-8, AMPI, Israel).

### Laser speckle flowgraphy

Spatiotemporal CBF was monitored using laser speckle flowgraphy (LSFG-Micro, Softcare, Fukuoka, Japan). An 830-nm laser diode was selected as a light source for the flow imaging because this wavelength of light likely has no effect on the ChR2(C128S), as the irradiated wavelength is far from its excitation range^44^. While the skull or exposed cortex was continuously illuminated, a speckle pattern was captured at a rate of 30 frames per sec (i.e., an exposure time of 33 ms in each frame), using a charge-coupled device (CCD) camera (600 × 480 pixels) attached to a microscope (SZ61TR, Olympus Corporation, Tokyo, Japan). The field of view was either 4.9 mm × 4.7 mm or 2.4 mm × 2.3 mm at 2-fold or 4-fold magnification of the objective lens (NA = 0.071 and a working distance of 110 mm), respectively (see Fig. 1A). The raw intensity image was converted to a flow image by calculating the mean blur rate (MBR) of each pixel using 3-consecutive frames^45^. The pre-stimulation baseline images were acquired for 30 sec before induction of photostimulation in each recording, and the post-stimulation images were acquired for at least 90 sec depending on the duration of the evoked changes in CBF. In each animal, 1–8 trials (average 3 trials per stimulation) were repeated for the same paradigms of the photostimulation with an onset-to-onset interval of >2 min.

### Pharmacology

To apply the drugs to the stimulated cortex, a section of skull approximately 3 mm in diameter was removed (N = 10 ChR2 mice), and the dura located near the edge of the opened area was carefully removed. Both the left and right hemispheres were opened in five out of ten animals to test a single drug in respective hemispheres. First, saline was applied to the opened area of the cortical surface, and the CBF response to photostimulation was measured with LSFG (i.e., pre-treatment control measurement). Then, a K^+^ channel inhibitor BaCl_2_ (0.1–0.5 mM in saline; Yoneyama Yakuhin Kogyo Co., Ltd., Japan), the non-selective COX inhibitor sodium indomethacin (0.5 mM in saline; INDACIN IV; MSD K.K., Japan), or voltage gated sodium channel inhibitor tetrodotoxin (TTX; 20–50 μM in saline; Nacalitesque, Kyoto, Japan) were topically applied (0.1–0.3 mL) to the overall exposed cortical surface. The concentration of the drugs used in the present study was determined according to previous studies^3^,^30^,^31^,^46^. At 20–60 min after drug administration, the photostimulation-induced CBF response was evaluated with the same protocols used for the pre-treatment control measurements. In this experiment, a stimulation power of the blue laser irradiation was adjusted so that the evoked CBF does not spread out of the field of view under pre-treatment conditions.

### Histology

At the end of the experiments, SR101 (10 mM in saline, Sigma, Japan), a marker of astrocytes/olligodendrocytes^47^,^48^, was intraperitoneally injected (0.10–0.15 mL, N = 6 ChR2 mice). At 3–4 hours after the injection, the brain was removed and fluorescently visualised to verify the expression of ChR2(C128S)-EYFP in SR101-labelled astrocytes using two-photon microscopy (TCS SP5MP, Leica Microsystems)^49^. Note that ChR2(C128S)-EYFP is expressed throughout the cell membrane including the astrocytic fine processes. The astrocytic expression of ChR2(C128S)-EYFP was dense in the parenchyma of the measured somatomotor cortex, but the astrocytes in the subsurface layers only sparsely expressed the transgene (Fig. 1B).

### Data analysis

A region of interest (ROI) was manually placed at the centre of the maximum response area for the LSFG flow images, and the mean time-course of the relative CBF changes normalised to the baseline averaged over 10-sec a pre-stimulation period were calculated by averaging the pixel values within the ROI. For comparisons of the CBF responses between laser-irradiated cortex (i.e., ipsilateral cortex) and unirradiated cortex (i.e., contralateral cortex), two ROIs (1 mm in diameter) were located at an activation foci in the ipsilateral cortex and an area contralateral to the activation foci in the other cortex (see Fig. 1A). Then, the response magnitude of the CBF was calculated by averaging CBF changes over 5 to 15 sec after the onset of photostimulation within the ROI. For the LSFG through the intact skull, it was shown that evoked changes in CBF were influenced by a large static scattering^50^. In this study, we did not correct any static components that may cause a slight underestimation of the CBF response magnitude.

Two-points of the onset time of the CBF response were defined. One is the time at which the relative CBF surpassed the mean plus 2-fold standard deviation of the pre-stimulation baseline, and the other one is the relative CBF surpassed the mean plus 3-fold standard deviation. For this particular analysis, a low-pass filter (<0.5 Hz) for time-course data in each pixel and a Gaussian filter for imaging data of the onset map across the pixels were applied. To characterise the spatial propagation of the CBF responses, the average onset times at pixels located 0.25 mm, 0.50 mm, and 0.75 mm from the centre of the activation were measured. The apparent propagation time was calculated by dividing the difference of the mean onset time between the target pixels (located on a circle of 0.25 mm, 0.5 mm, and 0.75 mm in radius around the activation foci) by a distance of 0.25 mm. The areas of activation induced by photostimulation were calculated as the number of pixels that exhibited a CBF response of more than 50% of the maximum response observed within the field of view.

Data are represented as means ± standard deviation in the animals measured. The statistical significance (*P* < 0.05) for comparisons between two strains of mice was determined with Student’s t-test, and a paired t-test was used for the comparisons of the ipsilateral and contralateral responses and the pharmacological tests (control vs. treatment). Dunnett’s test was also used to evaluate the propagated response onset time from the activation foci.

## Additional Information

**How to cite this article**: Masamoto, K. *et al.* Unveiling astrocytic control of cerebral blood flow with optogenetics. *Sci. Rep.* **5**, 11455; doi: 10.1038/srep11455 (2015).

## Supplementary Material

Supplementary Information

## References

[b1] Zonta M. *et al.* Neuron-to-astrocyte signaling is central to the dynamic control of brain microcirculation. Nat. Neurosci. 6, 43–50 (2003). 12469126 10.1038/nn980

[b2] Mulligan S. J. & MacVicar B. A. Calcium transients in astrocyte endfeet cause cerebrovascular constrictions. Nature. 431, 195–199 (2004). 15356633 10.1038/nature02827

[b3] Takano T. *et al.* Astrocyte-mediated control of cerebral blood flow. Nat. Neurosci. 9, 260–267 (2006). 16388306 10.1038/nn1623

[b4] Zlokovic B. V. Neurovascular pathways to neurodegeneration in Alzheimer’s disease and other disorders. Nat. Rev. Neurosci. 12, 723–738 (2011). 22048062 10.1038/nrn3114 PMC4036520

[b5] Metea M. R. & Newman E. A. Glial cells dilate and constrict blood vessels: a mechanism of neurovascular coupling. J. Neurosci. 26, 2862–2870 (2006). 16540563 10.1523/JNEUROSCI.4048-05.2006 PMC2270788

[b6] Gordon G. R. *et al.* Brain metabolism dictates the polarity of astrocyte control over arterioles. Nature. 456, 745–749 (2008). 18971930 10.1038/nature07525 PMC4097022

[b7] Filosa J. A. *et al.* Local potassium signaling couples neuronal activity to vasodilation in the brain. Nat. Neurosci. 9, 1397–1403 (2006). 17013381 10.1038/nn1779

[b8] Girouard H. *et al.* Astrocytic endfoot Ca2+ and BK channels determine both arteriolar dilation and constriction. Proc. Natl. Acad. Sci. U. S. A. 107, 3811–3816 (2010). 20133576 10.1073/pnas.0914722107 PMC2840528

[b9] Attwell D. *et al.* Glial and neuronal control of brain blood flow. Nature. 468, 232–243 (2010). 21068832 10.1038/nature09613 PMC3206737

[b10] Petzold G. C. & Murthy V. N. Role of astrocytes in neurovascular coupling. Neuron. 71, 782–797 (2011). 21903073 10.1016/j.neuron.2011.08.009

[b11] Howarth C. The contribution of astrocytes to the regulation of cerebral blood flow. Front. Neurosci. 8, 103 (2014). 24847203 10.3389/fnins.2014.00103 PMC4023041

[b12] Schummers J., Yu H. & Sur M. Tuned responses of astrocytes and their influence on hemodynamic signals in the visual cortex. Science. 320, 1638–1643 (2008). 18566287 10.1126/science.1156120

[b13] Calcinaghi N. *et al.* Metabotropic glutamate receptor mGluR5 is not involved in the early hemodynamic response. J. Cereb. Blood. Flow. Metab. 31, e1-10 (2011). 21731033 10.1038/jcbfm.2011.96 PMC3185891

[b14] Lind B. L. *et al.* Rapid stimulus-evoked astrocyte Ca2+ elevations and hemodynamic responses in mouse somatosensory cortex *in vivo*. Proc. Natl. Acad. Sci. U. S. A. 110, E4678–4687 (2013). 24218625 10.1073/pnas.1310065110 PMC3845114

[b15] Nizar K. *et al.* *In vivo* stimulus-induced vasodilation occurs without IP3 receptor activation and may precede astrocytic calcium increase. J. Neurosci. 33, 8411–8422 (2013). 23658179 10.1523/JNEUROSCI.3285-12.2013 PMC3712855

[b16] Takata N. *et al.* Cerebral blood flow modulation by Basal forebrain or whisker stimulation can occur independently of large cytosolic Ca2+ signaling in astrocytes. PLoS. One. 8, e66525 (2013). 23785506 10.1371/journal.pone.0066525 PMC3681769

[b17] Bonder D. E. & McCarthy K. D. Astrocytic Gq-GPCR-Linked IP3R-Dependent Ca2+ Signaling Does Not Mediate Neurovascular Coupling in Mouse Visual Cortex *In Vivo*. J. Neurosci. 34, 13139–13150 (2014). 25253859 10.1523/JNEUROSCI.2591-14.2014 PMC4172806

[b18] Jego P., Pacheco-Torres J., Araque A. & Canals S. Functional MRI in mice lacking IP3-dependent calcium signaling in astrocytes. J. Cereb. Blood. Flow. Metab. 34, 1599–1603 (2014). 25099754 10.1038/jcbfm.2014.144 PMC4269735

[b19] Otsu Y. *et al.* Calcium dynamics in astrocyte processes during neurovascular coupling. Nat. Neurosci. 18, 210–219 (2015). 25531572 10.1038/nn.3906 PMC4651918

[b20] Sun W. *et al.* Glutamate-dependent neuroglial calcium signaling differs between young and adult brain. Science. 339, 197–200 (2013). 23307741 10.1126/science.1226740 PMC3569008

[b21] Sofroniew M. V. & Vinters H. V. Astrocytes: biology and pathology. Acta. Neuropathol. 119, 7–35 (2010). 20012068 10.1007/s00401-009-0619-8 PMC2799634

[b22] Nagel G. *et al.* Channelrhodopsin-2, a directly light-gated cation-selective membrane channel. Proc. Natl. Acad. Sci. U. S. A. 100, 13940–13945 (2003). 14615590 10.1073/pnas.1936192100 PMC283525

[b23] Tanaka K. F. *et al.* Expanding the repertoire of optogenetically targeted cells with an enhanced gene expression system. Cell. Rep. 2, 397–406 (2012). 22854021 10.1016/j.celrep.2012.06.011

[b24] Yizhar O. *et al.* Optogenetics in neural systems. Neuron. 71, 9–34 (2011). 21745635 10.1016/j.neuron.2011.06.004

[b25] Figueiredo M. *et al.* Optogenetic experimentation on astrocytes. Exp. Physiol. 96, 40–50 (2011). 21041318 10.1113/expphysiol.2010.052597

[b26] Sasaki T. *et al.* Application of an optogenetic byway for perturbing neuronal activity via glial photostimulation. Proc. Natl. Acad. Sci. U. S. A. 109, 20720–20725 (2012). 23185019 10.1073/pnas.1213458109 PMC3528589

[b27] Koehler R. C., Roman R. J. & Harder D. R. Astrocytes and the regulation of cerebral blood flow. Trends. Neurosci. 32, 160–169 (2009). 19162338 10.1016/j.tins.2008.11.005

[b28] Niwa K. *et al.* Cyclooxygenase-2 contributes to functional hyperemia in whisker-barrel cortex. J. Neurosci. 20, 763–770 (2000). 10632605 10.1523/JNEUROSCI.20-02-00763.2000 PMC6772412

[b29] Lecrux C. *et al.* Pyramidal neurons are “neurogenic hubs” in the neurovascular coupling response to whisker stimulation. J. Neurosci. 31, 9836–9847 (2011). 21734275 10.1523/JNEUROSCI.4943-10.2011 PMC6703330

[b30] Knot H. J., Zimmermann P. A. & Nelson M. T. Extracellular K+ -induced hyperpolarizations and dilatations of rat coronary and cerebral arteries involve inward rectifier K+ channels. J. Physiol. (Lond.) 492, 419–430 (1996). 9019539 10.1113/jphysiol.1996.sp021318 PMC1158837

[b31] Sobey C. G. Potassium channel function in vascular disease. Arterioscler. Thromb. Vasc. Biol. 21, 28–38 (2001). 11145930 10.1161/01.atv.21.1.28

[b32] Dunn A. K. Laser speckle contrast imaging of cerebral blood flow. Ann. Biomed. Eng. 40, 367–377 (2012). 22109805 10.1007/s10439-011-0469-0 PMC3288249

[b33] Beppu K. *et al.* Optogenetic countering of glial acidosis suppresses glial glutamate release and ischemic brain damage. Neuron. 81, 314–320 (2014). 24462096 10.1016/j.neuron.2013.11.011

[b34] Fergus A. & Lee K. S. Regulation of cerebral microvessels by glutamatergic mechanisms. Brain. Res. 754, 35–45 (1997). 9134957 10.1016/s0006-8993(97)00040-1

[b35] Morley P. *et al.* Evidence that functional glutamate receptors are not expressed on rat or human cerebromicrovascular endothelial cells. J. Cereb. Blood. Flow. Metab. 18, 396–406 (1998). 9538905 10.1097/00004647-199804000-00008

[b36] Dunn K. M. & Nelson M. T. Potassium channels and neurovascular coupling. Circ. J. 74, 608–616 (2010). 20234102 10.1253/circj.cj-10-0174 PMC4405141

[b37] Masamoto K. & Kanno I. Anesthesia and the quantitative evaluation of neurovascular coupling. J. Cereb. Blood. Flow. Metab. 32, 1233–1247 (2012). 22510601 10.1038/jcbfm.2012.50 PMC3390804

[b38] Odette L. L. & Newman E. A. Model of potassium dynamics in the central nervous system. Glia. 1, 198–210 (1988). 2976039 10.1002/glia.440010305

[b39] Chuquet J., Hollender L. & Nimchinsky E. A. High-resolution *in vivo* imaging of the neurovascular unit during spreading depression. J. Neurosci. 27, 4036–4044 (2007). 17428981 10.1523/JNEUROSCI.0721-07.2007 PMC6672520

[b40] Chen B. R. *et al.* High-speed vascular dynamics of the hemodynamic response. Neuroimage. 54, 1021–1030 (2011). 20858545 10.1016/j.neuroimage.2010.09.036 PMC3018836

[b41] Dietrich H. H., Kajita Y. & Dacey R. G. Jr. Local and conducted vasomotor responses in isolated rat cerebral arterioles. Am. J. Physiol. 271, H1109–1116 (1996). 8853348 10.1152/ajpheart.1996.271.3.H1109

[b42] Paulson O. B. & Newman E. A. Does the release of potassium from astrocyte endfeet regulate cerebral blood flow? Science. 237, 896–898 (1987). 3616619 10.1126/science.3616619 PMC2505270

[b43] Metea M. R., Kofuji P. & Newman E. A. Neurovascular coupling is not mediated by potassium siphoning from glial cells. J. Neurosci. 27, 2468–2471 (2007). 17344384 10.1523/JNEUROSCI.3204-06.2007 PMC2289782

[b44] Zhang F. *et al.* Circuit-breakers: optical technologies for probing neural signals and systems. Nat. Rev. Neurosci. 8, 577–581 (2007). 17643087 10.1038/nrn2192

[b45] Konishi N., Tokimoto Y., Kohra K. & Fujii H. New laser speckle flowgraphy system using CCD camera. Optical. Review. 9, 163–169 (2002).

[b46] Leithner C. *et al.* Pharmacological uncoupling of activation induced increases in CBF and CMRO2. J. Cereb. Blood. Flow. Metab. 30, 311–322 (2010). 19794398 10.1038/jcbfm.2009.211 PMC2949119

[b47] Nimmerjahn A., Kirchhoff F., Kerr J. N. & Helmchen F. Sulforhodamine 101 as a specific marker of astroglia in the neocortex *in vivo*. Nat. Methods. 1, 31–37 (2004). 15782150 10.1038/nmeth706

[b48] Hill R. A. & Grutzendler J. *In vivo* imaging of oligodendrocytes with sulforhodamine 101. Nat. Methods. 11, 1081–1082 (2014). 25357236 10.1038/nmeth.3140 PMC4539948

[b49] Masamoto K. *et al.* Repeated longitudinal *in vivo* imaging of neuro-glio-vascular unit at the peripheral boundary of ischemia in mouse cerebral cortex. Neuroscience. 212, 190–200 (2012). 22516017 10.1016/j.neuroscience.2012.03.034

[b50] Zakharov P. *et al.* Dynamic laser speckle imaging of cerebral blood flow. Opt. Express. 17, 13904–13917 (2009). 19654798 10.1364/oe.17.013904

