# Supplementary material for: Unveiling astrocytic control of cerebral blood flow with optogenetics
Source: Sci Rep. 2015 Jun 16;5:11455. doi: 10.1038/srep11455 (PMC4468581; doi:10.1038/srep11455)
Supplement: Supplementary Information [file srep11455-s1.doc]

**Unveiling astrocytic control of cerebral blood flow with optogenetics**

Kazuto Masamoto^a, b, c,*^, Miyuki Unekawa^d^, Tatsushi Watanabe^a^, Haruki Toriumi^d^,

Hiroyuki Takuwa^c^, Hiroshi Kawaguchi^c^, Iwao Kanno^c^, Ko Matsui^e^, Kenji F. Tanaka^f^,

Yutaka Tomita^d^, Norihiro Suzuki^d^

^a^Faculty of Informatics and Engineering, ^b^Brain Science Inspired Life Support Research Centre, University of Electro-Communications, 1-5-1 Chofugaoka, Chofu, Tokyo 182-8585, Japan; ^c^Molecular Imaging Centre, National Institute of Radiological Sciences, 4-9-1 Anagawa, Inage, Chiba 263-8555, Japan; ^d^Department of Neurology, ^f^Department of Neuropsychiatry, School of Medicine Keio University, 35 Shinanomachi, Shinjuku, Tokyo 160-8582, Japan; ^e^Division of Interdisciplinary Medical Science, Tohoku University Graduate School of Medicine, 2-1 Seiryomachi, Aoba, Sendai, Miyagi 980-8587, Japan

Supplementary

Figure S1 Reproducible response of CBF evoked by photostimulation.

Onset-to-onset interval of 120 sec was sufficient to reliably evoke the robust CBF response to the 3-sec photostimulation (0.03 mW blue laser). The CBF time-course data were averaged across the pixels within the activation focus (1 mm in diameter), measured with LSFG.

Figure S2 Effects of BaCl2 administration on the baseline neural activity and CBF in ChR2 mice.

Baseline neural activity was monitored with electroencephalography (EEG) with Ag/AgCl electrode (a tip diameter of 0.2 mm; EEG-5002Ag, Bioresearch Center Co., Ltd.), while CBF was concurrently monitored with LSFG. **A**) No detectable changes in the baseline EEG represented for α band (8-13Hz) and mean blur rate (MBR) values, measured for 10 sec in each condition, were observed across pre-treatment (left), and 10-min (center) to 30-min (right) after topical administration of BaCl_2_ (0.5 mM). The EEG electrode was placed near the edge of the opened cortex (left side in the image). The recording was conducted at 1kHz sampling rate and EEG potential was digital filtered with 8-13Hz, 13-30Hz, and 30-100Hz for α, β, and γ bands, respectively, as described elsewhere (Unekawa et al., 2012). A root mean square of EEG measurements obtained for 10 sec in each state of the experiments (e.g., pre-treatment controls and drug applications) was calculated. The LSFG image (bottom panels) represents baseline MBR averaged over 10 sec in each condition. The baseline value of MBR was calculated by averaging the values within the activation foci (0.5 mm in a diameter, represented as a circle in the panels) **B,C**) Population data consistently showed no significant differences in the baseline EEG activity and MBR values over the periods of pre-treatment, 10-min and 30-min after the BaCl_2_ administration (p > 0.05; Dunnett's test, N = 4 animals).

Figure S3 Photostimulation-induced vasodilation measured by using two-photon microscopy.

The cortical vasculature was labeled with sulforhodamine 101 (10 μM in saline), and the image was obtained with two-photon microscopy excited at 900 nm. The cortical vessels significantly dilates (p = 0.002) after 3-sec blue light photostimuation; 45.4 ± 0.4 μm vs. 49.1 ±1.3 μm before (left) vs. after (right) induction of photostimulation, respectively (N = 1 animal). The results indicate that CBF increases observed after photostimulation was due to vasodilation driven by the activated astrocytes.

References

Unekawa, M., Tomita, M., Tomita, Y., Toriumi, H., Suzuki, N., Sustained decrease and remarkable increase in red blood cell velocity in intraparenchymal capillaries associated with potassium-induced cortical spreading depression. *Microcirculation.* **19**:166-174 (2012)
